# Saliency guided data augmentation strategy for maximally utilizing an object’s visual information

**DOI:** 10.1371/journal.pone.0274767

**Published:** 2022-10-13

**Authors:** Junhyeok An, Soojin Jang, Junehyoung Kwon, Kyohoon Jin, YoungBin Kim

**Affiliations:** Department of Image Science and Arts, Chung-Ang University, Dongjak, Seoul, Korea; Hanyang University, KOREA, REPUBLIC OF

## Abstract

Among the various types of data augmentation strategies, the mixup-based approach has been particularly studied. However, in existing mixup-based approaches, object loss and label mismatching can occur if random patches are utilized when constructing augmented images, and additionally, patches that do not contain objects might be included, which degrades performance. In this paper, we propose a novel augmentation method that mixes patches in a non-overlapping manner after they are extracted from the salient regions in an image. The suggested method can make effective use of object characteristics, because the constructed image consists only of visually important regions and is robust to noise. Since the patches do not occlude each other, the semantically meaningful information in the salient regions can be fully utilized. Additionally, our method is more robust to adversarial attack than the conventional augmentation method. In the experimental results, when Wide ResNet was trained on the public datasets, CIFAR-10, CIFAR-100 and STL-10, the top-1 accuracy was 97.26%, 83.99% and 82.40% respectively, which surpasses other augmentation methods.

## 1 Introduction

Deep neural networks have demonstrated excellent performance on visual recognition tasks, such as image classification, object detection, and segmentation [1–3]. Neural networks can be extremely flexible, due to the large number of parameters involved. However, this feature may cause overfitting to a training dataset’s distribution, and overfitting has a negative effect on previously unseen data.

To enhance the generalization ability of neural networks, various regularization methods have been explored. In recent studies, many data augmentation strategies have been developed, including removing part of an image, mixing two or more images [4–7], using mesh-like masks [8] or generating data using neural networks [9]. The data augmentation methods that use two or more images [6, 7] generally select background regions at random and blend them to construct an augmented image. However, if random patches are utilized to construct augmented images, patches that do not contain objects might be included, which in turn degrades performance. To alleviate this problem, a study using saliency maps [10–12] was conducted. A saliency map is an image that highlights a region on which the human eye focuses [13]. The data augmentation methods that use saliency maps extract semantically important regions from these maps as a patch. Through this process, semantically meaningful regions can be utilized to train the network. However, when blending the patches, the patch can overlap the source image and some areas of the object become occluded. Therefore, the network may not be able to learn semantically meaningful regions.

In this study, we propose an augmentation method that utilize the saliency maps from multiple images to allow all image parts to contain information about the semantically important regions, which are blended in a non-overlapping manner. In the proposed technique, after the saliency maps are extracted from each image, the semantically important regions are extracted in the form of patches, which include the map’s peak parts. An augmented image is then constructed based on the four extracted patches and the patches are labeled according to the proportions of the four areas related to each patch. In the proposed method, perturbation of the input data is avoided, since only the information about each image object is used during data augmentation. Our proposed method extracts the most salient region in the image and blends it to prevent the network from learning irrelevant features. In addition, semantically meaningful regions can be extracted and blended in various ways to make the network more robust. Furthermore, our proposed method makes the network more robust to adversarial attack, which causes images to be misclassified by the network.

The proposed method demonstrated top-1 accuracies of 97.26, 83.99, and 82.40% on the CIFAR-10, CIFAR-100, and STL-10 datasets, respectively [14, 15]; this performance is better than that reported for any other data augmentation method. When experiencing an adversarial attack using the fast gradient sign method (FGSM) [16], the proposed method demonstrated higher top-1 accuracies than the baseline models on the CIFAR-10 and CIFAR-100 datasets by 19.34% and 6.87%, respectively, which indicates the robustness of the proposed data augmentation method.

## 2 Related work

### 2.1 Mixed data augmentation

Data augmentation has been used to improve model generalization by alleviating overfitting [17, 18]. Data augmentation methods that use simple image manipulation apply color space transformations such as RGB shift and contrast transformation, or geometric transformations such as distortion and flipping [17].

To improve model performance, researchers have also developed a data augmentation method that generates a new image with a mixed technique that uses a subset of the training images.

In the mixup method [5], a network is trained to learn two image samples using a convex combination of the images and their corresponding labels. Unlike the conventional method, which only predicts one class, the mixup method predicts two different classes, which produces smoother estimation and margin maximization. The mixup method may be applied to a feature space after convolution, and it is then called the Manifold Mixup method [19]. In the CutMix method [6], a patch is randomly copied from one input image and pasted onto another image, which provides the model with enhanced localization and classification abilities. A method for mixing feature maps using multiple inputs and outputs has also been proposed [20]. Another method known as ResizeMix [21] resizes and mixes the patch to preserve the information in the image.

To obtain more information, methods for mixing various images have also been proposed. In the random image cropping and patching(RICAP) method [7], patches are randomly extracted from four training images to generate a new image. In addition, a method for mixing four images to improve performance for object detection tasks has been proposed [22]. Another method, the Recursive Mix method [23] continuously reuses augmented data from previous iterations.

The proposed method also uses four images to generate a new image, but it differs from the existing methods in that it uses a saliency map to generate the new image, which contains only semantically important regions.

### 2.2 Mixed data augmentation using semantically important regions

Since the extraction of random regions during the process of data augmentation mixing may incur data loss, researchers have developed augmentation methods in which semantically important regions are retained during mixing. Attentive CutMix [24] extracts attentive regions using a separate pre-trained network and then mixes them. Additional methods for extracting regions with semantic importance utilizing class activation map have been developed [25]. The Fmix method [26] uses a binary mask that is obtained by applying a threshold to low-frequency images sampled from a Fourier space. By using the attention map generated by the transformer structure, which has lately been utilized widely in many tasks, the TransMix method [27] suggests another method for extracting the semantically important regions.

Several authors have proposed an augmentation method in which the semantically important regions in images are obtained using a saliency map. In the KeepAugment method [28] the weights from a previously learned model are used to detect the saliency map, and augmentation is performed using the detected saliency map to preserve the corresponding regions. KeepAugment has an advantage because it includes semantically important regions without generating noisy augmented examples. The Saliency Mix method [10] extracts semantically important regions in the form of patches from a source image, and mixes them with the target image. In a variant of the mixup method called the Puzzle Mix method [11], the mixup performance is enhanced by the use of a saliency map. The original mixup method may generate incorrect data and labels, due to the absence of key information. To overcome these problems, the Puzzle Mix method uses statistical methods and saliency information. In the SuperMix method [12], an image is mixed based on salient regions by using the teacher’s knowledge. In this paper, we propose a method in which robust augmentation is ensured by using the saliency information from four images. The Co-Mixup method [29] proposes mixing images based on salient regions by finding the best combination among image collections.

In this study, we propose a simple but effective data augmentation method. The saliency map for the four images is used to extract semantically significant regions, which are then mixed in a non-overlapping manner to minimize information loss on the source image.

## 3 Methods

**Fig 1:**
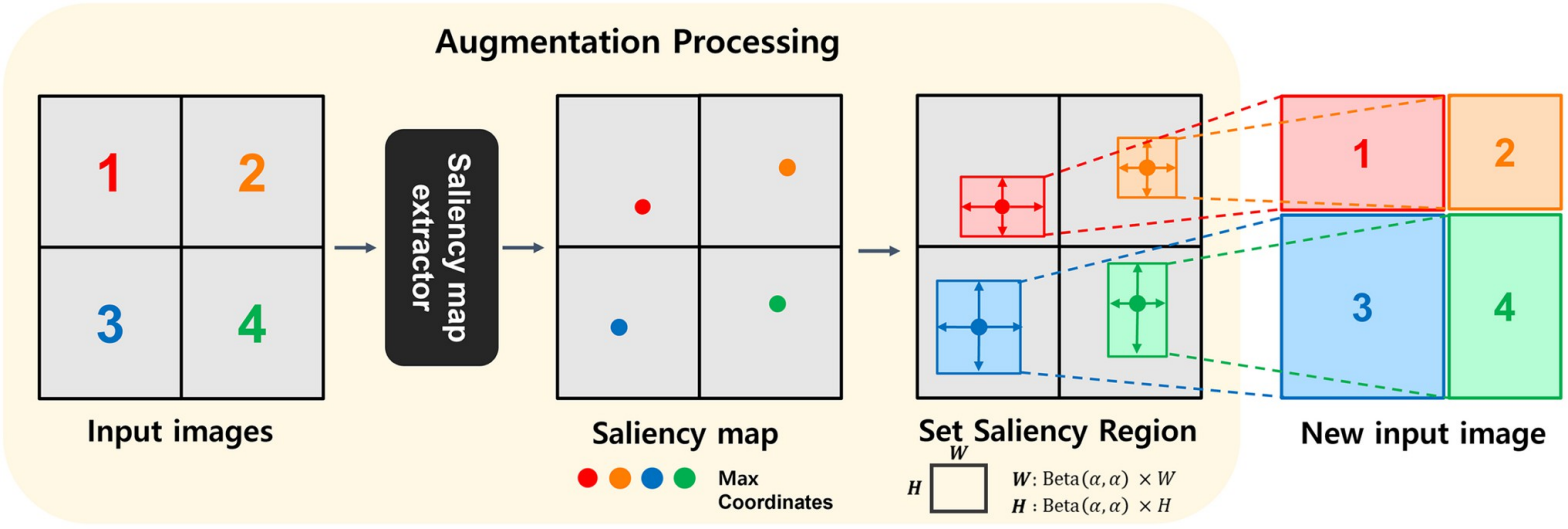
An overview of the proposed data augmentation method.

We propose a data augmentation method that constructs a new image as a semantically important region-based patch, using a saliency map for model generalization. Using the saliency map based on the four training images, each patch is extracted based on the object area, and a new image composed of only that patch is created. The labels are also adjusted according to the proportion of the area occupied by each patch. The proposed method is shown in Fig 1

### 3.1 Saliency map extractor

The proposed data augmentation approach involves the extraction of semantically important regions from all training images. To construct an augmented image, *n* images are randomly selected from the training image, and we use four images(*n* = 4) in the proposed method. The width and height of the source image are expressed as *W*, *H*.

$$\begin{array}{c} I_{n}^{sm}=s\left(I_{n}\right)\;,for\;n\in \left\{1,2,3,4\right\} \end{array}$$ (1)

*I*_*n*_ is a randomly selected training image, from which a patch is extracted. When extracting patches, a saliency map is used. $I_{n}^{sm}$ is the saliency map of image *I*_*n*_, and *s*(⋅) is a grained saliency map detection function that uses an OpenCV library to extract a grained saliency map [30].

### 3.2 Determination of semantically important regions

We extract the selected 4 image patches *n* ∈ {1, 2, 3, 4}, and blend them to occupy the upper left, upper right, lower left, and lower right regions, respectively.

$$\begin{array}{c} U_{n},\;V_{n}=argmax\left(I_{n}^{sm}\right)\;,for\;n\in \left\{1,2,3,4\right\} \end{array}$$ (2)

The proposed data augmentation method shown in Fig 1 constructs a new image using four images. *U*_*n*_ and *V*_*n*_ are the coordinates of the peak point that has the highest value obtained from the saliency map, which is the semantically important region. The patch region is determined so that *U*_*n*_ and *V*_*n*_ become the patch’s center coordinates, and the width and height of the cropped patch *n* are as follows.

$$\begin{array}{c} \begin{array}{c} w_{1}=w_{3}=W*Uniform\left(0,1\right)\\ h_{1}=h_{3}=H*Uniform\left(0,1\right)\\ w_{2}=w_{4}=W-w_{1}\\ h_{2}=h_{4}=H-h_{1} \end{array} \end{array}$$ (3)

The sizes of the upper left image *w*_1_, *h*_1_ are multiplied by the number extracted from the uniform distribution by the original image sizes *W*, *H*, and the size of the upper right, lower left, and lower right coordinates are determined using the same process. The sizes of each image and the coordinates of the saliency map’s points with the highest value are used to set the semantically important regions of each image, which are then extracted in the form of patches.

### 3.3 Image mixing and label mixing

An augmented image is formed by blending the patches in a non-overlapping manner. The newly generated image is defined as follows.

$$\begin{array}{c} y_{sm}=\sum_{n=1}^{4}\frac{w_{n}h_{n}}{WH}\cdot y_{n} \end{array}$$ (4)

The *y*_*sm*_ label of a newly constructed image is set by producing the label *y*_*n*_ for each image and cropping it according to the size of the four cropped images. The loss function for learning the newly constructed image is calculated as follows

$$\begin{array}{c} Loss=\sum_{i=1}^{N}ylog\left(\hat{y_{i}}\right) \end{array}$$ (5)

where $\hat{y}$ is the value predicted by the model.

In summary, the proposed data augmentation method generates a new image using only the semantically important regions in the four images, which are used for network learning. The new image’s label is set by producing label *y*_*n*_ for each image, and the labels are then cropped according to the size of the four cropped images.

## 4 Experiments

### 4.1 Implementation details

#### 4.1.1 Classification

To investigate the proposed data augmentation method’s performance, we used three public datasets. The CIFAR and STL-10 datasets [14, 15] were used, and the ResNet [31] and WideResNet [32] architectures were applied. The CIFAR-10 and CIFAR-100 datasets consist of 60,000 color images (50,000 training images and 10,000 test images), where each image size is 32 × 32. The STL-10 dataset consists of 13,000 labeled images (5,000 training images and 8,000 test images), where each image size is 96 × 96. We trained the networks for 300 epochs with a batch size of 64. The optimizer used for ResNet and WideResNet learning was stochastic gradient descent, and learning was performed with a learning rate of 0.25, a momentum of 0.9, and a weight decay of 1e-4. The learning rate decreased by a factor of 0.2 after 60, 120, 160, and 200 epochs.

The proposed data augmentation method was compared with other methods and a basic augmentation method was developed by applying random crops and random horizontal flips to the baseline model. In the cutout method [4] the number of holes was set to 1, and the hole lengths were set to 8 and 16 for CIFAR-10 and CIFAR-100, respectively. The *α* values of the beta distributions used for data augmentation in CutMix [6], RICAP [7], and SaliencyMix [10] were set to 1.0.

#### 4.1.2 Adversarial attack

FGSM [16] was used to evaluate the method’s robustness against adversarial attack. The experiments were performed using a non-targeted attack with an added perturbation of 0.03 and one iteration, on the ResNet-50 architecture, which was trained on the public dataset, CIFAR.

### 4.2 CIFAR classification

The method proposed in Table 1 required more training time than the other methods because, unlike other generalization methods, the object region is extracted from the saliency map, which is composed of all of the training images. However, the time required for testing was the same for the proposed method as that for the other generalization methods. The results show that the proposed method performed better than the other generalization methods, because the object information was maximized by extracting the object regions for all of the training images.

**Table 1:** Training times of the compared methods on the CIFAR dataset.

Model	ResNet-50	CutMix	RICAP	SaliencyMix	Ours
time(s)	0.041	0.042	0.046	0.042	0.079

Table 2 shows the results for the five augmentation methods when using the WideResNet-32 architecture, and Table 3 shows the results of the experiments that evaluated seven augmentation methods with the ResNet-50 and ResNet-101 architectures on the CIFAR dataset. The proposed method had higher performance than the baseline and other existing data augmentation methods when using all of the tested architectures.

**Table 2:** Classification performance of the Wide ResNet-32 architectures on the CIFAR-10 and CIFAR-100 datasets.

Augmentation	CIFAR-10 top-1 accuracy	CIFAR-100 top-1 accuracy
WideResNet-32	92.70%	72.80%
+ Augmentation	96.31%	80.38%
+ CutMix	97.18%	83.52%
+ SaliencyMix	97.18%	83.56%
+ Ours	**97.26%**	**83.99%**

**Table 3:** Comparison of the classification performance by the ResNet-50 and ResNet-101 architectures on the CIFAR-10 and CIFAR-100 datasets.

Augmentation	CIFAR-10 top-1 accuracy	CIFAR-100 top-1 accuracy
ResNet-50	90.62%	70.02%
+ Augmentation	93.22%	73.71%
+ Cutout	94.36%	74.83%
+ RICAP	93.42%	74.78%
+ CutMix	94.22%	75.64%
+ SaliencyMix	94.19%	74.49%
+ Ours	**94.47%**	**75.67%**
ResNet-101	91.65%	71.18%
+ Augmentation	93.99%	74.76%
+ Cutout	94.97%	74.49%
+ RICAP	94.93%	75.47%
+ CutMix	95.48%	77.29%
+ SaliencyMix	94.88%	75.35%
+ Ours	**95.51%**	**78.51%**

The top-1 accuracy of the proposed method on the CIFAR-10 dataset was better than that of the ResNet-50, ResNet-101, and WideResNet-32 architectures by 3.85%p, 3.86%p, and 4.56%p, respectively, and in the case of the CIFAR-10 dataset, the improvements were 5.65%p, 7.33%p, and 11.19%p, respectively. Compared with RICAP, the proposed method uses four images, but the proposed method produced a 0.52–3.04%p higher top-1 accuracy. It seems that the method of extracting salient regions and constructing patches allows the network to learn more semantically meaningful features than RICAP, whose data may not contain objects. Compared with SaliencyMix, which uses information from semantically important regions of the saliency map, the top-1 accuracy of the proposed method was higher by 0.08–3.16%p. Blending the four images in a non-occluded manner seems to be more effective than SaliencyMix, where a specific image region is occluded by a patch.

### 4.3 STL-10 classification

We trained using the pubic dataset, STL-10, to determine whether our method works well even for small datasets. Table 4 shows the results of six augmentation methods using the WideResNet-16 architecture on the STL-10 dataset. These results confirm that the proposed method’s performance is better than that of the baseline as well as the other data augmentation methods. The proposed method’s accuracy improved in terms of the top-1 list by 13.85% on the STL-10 dataset, and the magnitude of improvement was largeron the STL-10 dataset than on the CIFAR dataset. Since a larger image is likely to contain information that is not an actual object in an image such as background information, the proposed method is more effective, in that it can learn using only information about the object, while including minimal information about the background.

**Table 4:** Classification performance of the WideResNet-16 architecture on the STL-10 dataset.

Augmentation	STL-10 top-1 accuracy
WideResNet-16	68.55%
+ Augmentation	80.43%
+ RICAP	81.13%
+ CutMix	81.92%
+ SaliencyMix	81.32%
+ Ours	**82.40%**

### 4.4 Number of images used for data augmentation

The proposed method constructs a new image by using the semantically important regions in four images so we additionally compared the performance achieved using different numbers of images for data augmentation. The CIFAR dataset was used for the experiment, and the images were resized to 224. Data augmentation was performed using two, four, and eight images (Table 5). The best performance was obtained when data augmentation was performed using four images this number minimized the amount of included background information, and maximized the usage of the information about the object. When eight images were used, only a part of the information about the object could be used, and there may therefore have been a part of the image that could not be learned. We therefore used the semantically important regions of four images in further work.

**Table 5:** CIFAR dataset classification experiment results according to the number of images used for data augmentation.

Number of images	CIFAR 10	CIFAR 100
2	**94.09%**	76.09%
4	**94.29%**	76.69%
8	**92.43%**	72.00%

### 4.5 Image-level augmentation vs. feature map-level augmentation

Although most data augmentation methods perform augmentation against images, some researchers have applied data augmentation to model feature map [19, 33], although they did not use a saliency map. We performed data augmentation at the image level against semantically important regions from the feature map by extracting the saliency map from the feature map $F\in R^{N\times W_{f}\times H_{f}\times C_{f}}$. The data augmentation procedure applied to the feature map was identical to that used for the training images.

$$\begin{array}{c} F_{n}^{sm}=s\left(F_{n}\right)\;for\;n=1\;\ldots \;N \end{array}$$ (6)

Data augmentation was performed by detecting the saliency map, *F*^*sm*^, of the feature map, *F*, for a subset of model layers, and the semantically important regions were identified.

To compare the results of augmentation using the proposed method with those for the feature map, the ResNet architecture was used on the CIFAR-10 and CIFAR-100 datasets. The data augmentation experiment for the feature map was performed with the feature map from the second ResNet-50 layer, as shown in Tables 6 and 7. The proposed method performed better when it was applied to a feature map than when it was applied to the input image.

**Table 6:** Augmentation performance using the ResNet architecture on the CIFAR-10 dataset for classification against image and feature maps.

Model	Image-level augmentation	Feature map-level augmentation
ResNet-18	**91.95%**	91.19%
ResNet-50	**94.47%**	94.46%
ResNet-101	**95.42%**	94.33%

**Table 7:** Augmentation performance using the ResNet architecture on the CIFAR-100 dataset when classifying the image and feature maps.

Model	Image-level augmentation	Feature map-level augmentation
ResNet-18	67.16%	**67.24**%
ResNet-50	**75.53%**	74.28%
ResNet-101	**78.51%**	76.27%

### 4.6 Robustness against adversarial attack

Adversarial attack [16, 34] lead machine learning models to misclassify the adversarial examples generated by intentionally adding minor perturbations to an image. Data augmentation has been used to respond to these adversarial attacks [10, 35] since it enhances a deep learning model’s capacity to address these attacks when the model is trained with adversarial training data. We compared the robustness of existing data augmentation methods against adversarial attack with that of the proposed method. The results of the experiments, which used FGSM [16] adversarial attack and applied the ResNet-50 model to the CIFAR datasets showed that the proposed method demonstrated higher top-1 accuracy than the baseline model by 19.34 and 6.87% for the CIFAR-10 and CIFAR-100 datasets, respectively. These results are shown in Table 8.

**Table 8:** Results of applying the ResNet-50 model trained on the CIFAR-10 and CIFAR-100 datasets using the FGSM method.

Augmentation	CIFAR-10 top-1 accuracy	CIFAR-100 top-1 accuracy
ResNet-50	42.02%	20.90%
+ Augmentation	50.00%	26.17%
+ RICAP	54.30%	26.88%
+ CutMix	56.65%	27.75%
+ SaliencyMix	51.22%	26.34%
+ Ours	**61.36%**	**27.77%**

### 4.7 Class activation map visualization

We examined a class activation map [36] produced by the model using the convolution neural network trained on the public dataset, STL-10. The models learned using different methods were compared with the STL-10 dataset baseline and existing data augmentation methods. The proposed method performs data augmentation using only information from semantically important regions of the saliency map, which includes information from each image, and is, unlike other methods or simple mixed methods that generate images using only two images. It therefore learns more about the features of the semantically important regions in the image than the baseline and existing data augmentation methods.

## 5 Conclusions

In this study we developed a data augmentation technique that uses the information from the semantically important regions in four images. Since the newly constructed image is composed only of the semantically important regions, the model is prevented from learning irrelevant features, such as the background. The proposed method was more robust against adversarial attacks than other data augmentation methods since it learns the features of these semantically important regions. The proposed data augmentation method exhibited top-1 accuracies of 97.26% and, 83.99% for the CIFAR-10 and, CIFAR-100 dataset, respectively. These percentages are higher than those of other data augmentation methods by 0.03–3.65%p. We also experimented with the STL-10 dataset to determine whether our method prevents overfitting and allows the model to effectively learn even on small datasets. In this experiment, it was also confirmed that our method works well on small datasets, leading to a 1.08-1.97%p performance improvement. In future work, we will test whether good performance is achieved not only on small data but also on large datasets, and we plan to study data augmentation techniques that are specialized for large datasets.
